# Puerarin Restores Autophagosome-Lysosome Fusion to Alleviate Cadmium-Induced Autophagy Blockade via Restoring the Expression of Rab7 in Hepatocytes

**DOI:** 10.3389/fphar.2021.632825

**Published:** 2021-04-14

**Authors:** Tao Wang, Li Wang, Yi Zhang, Jian Sun, Yilin Xie, Yan Yuan, Jianhong Gu, Jianchun Bian, Zongping Liu, Hui Zou

**Affiliations:** ^1^ College of Veterinary Medicine, Yangzhou University, Yangzhou, China; ^2^ Joint International Research Laboratory of Agriculture and Agri-Product Safety, School of Horticulture and Plant Protection, Yangzhou University, Yangzhou, China; ^3^ Jiangsu Co-innovation Center for Prevention and Control of Important Animal Infectious Diseases and Zoonoses, Yangzhou, China

**Keywords:** cadmium, puerarin, autophagy, autophagosome-lysosome fusion, Rab7, hepatocyte

## Abstract

Autophagic dysfunction is one of the main mechanisms by which the environmental pollutant cadmium (Cd) induces cell injury. Puerarin (Pue, a monomeric Chinese herbal medicine extract) has been reported to alleviate Cd-induced cell injury by regulating autophagy pathways; however, its detailed mechanisms are unclear. In the present study, to investigate the detailed mechanisms by which Pue targets autophagy to alleviate Cd hepatotoxicity, alpha mouse liver 12 (AML12) cells were used to construct a model of Cd-induced hepatocyte injury *in vitro*. First, the protective effect of Pue on Cd-induced cell injury was confirmed by changes in cell proliferation, cell morphology, and cell ultrastructure. Next, we found that Pue activated autophagy and mitigated Cd-induced autophagy blockade. In this process, the lysosome was further activated and the lysosomal degradation capacity was strengthened. We also found that Pue restored the autophagosome-lysosome fusion and the expression of Rab7 in Cd-exposed hepatocytes. However, the fusion of autophagosomes with lysosomes and autophagic flux were inhibited after knocking down *Rab7*, and were further inhibited after combined treatment with Cd. In addition, after knocking down *Rab7*, the protective effects of Pue on restoring autophagosome-lysosome fusion and alleviating autophagy blockade in Cd-exposed cells were inhibited. In conclusion, Pue-mediated alleviation of Cd-induced hepatocyte injury was related to the activation of autophagy and the alleviation of autophagy blockade. Pue also restored the fusion of autophagosomes and lysosomes by restoring the protein expression of Rab7, thereby alleviating Cd-induced autophagy blockade in hepatocytes.

## Introduction

Environmental heavy metal pollution, such as Cd pollution, is a global concern. The threat of Cd pollution in the environment to human health cannot be ignored because of the wide range of sources, the continuity of exposure to the environment, bioaccumulation, and multi-organ toxicity (Amadi et al., 2019; Genchi et al., 2020). Cd is a predisposing and promoting factor for the occurrence and development of many diseases. In addition to being associated with the occurrence of neurodegenerative diseases, cardiovascular diseases, cerebrovascular diseases, bone diseases, and cancer (Genchi et al., 2020), Cd exposure can also promote the development of musculoskeletal diseases and fatty liver (Kim et al., 2018; Reyes-Hinojosa et al., 2019), and aggravate diabetes and nephropathy (Hagedoorn et al., 2020). The liver is an important hub for many physiological processes, and acute or chronic liver diseases are prevalent worldwide (Trefts et al., 2017; Asrani et al., 2019). Unfortunately, the liver is one of the main target organs of Cd, and the problem of Cd pollution in the environment increases the burden of liver diseases worldwide. There have been numerous reports on the liver toxicity mechanism of Cd. Previous studies have shown that in addition to oxidative stress, apoptosis, and epigenetic changes, the destruction of autophagy is an important toxic mechanism of Cd (Genchi et al., 2020; Zou et al., 2020). These reports on the toxicity mechanism of Cd have provided a theoretical basis and research directions to cope with the effects of Cd on human and animal health.

Autophagy is the basic physiological process by which eukaryotic cells maintain homeostasis. Alterations in autophagy occur during many diseases. The lack or dysfunction of autophagy is closely related to the occurrence and development of many diseases, in which the regulation of autophagy plays an important role in their treatment (Mizushima et al., 2008; Condello et al., 2019). Targeting autophagy as a treatment strategy has been studied in many disease models, with significant progress. Targeting autophagy is considered to be a promising treatment strategy for tumors, cardiomyopathy, coronary heart disease, and pulmonary fibrosis disease (Lawrence and Nho, 2018; Dong et al., 2019; Zech et al., 2020). Furthermore, based on the important role of the autophagy pathway in viral infection, Yang et al. proposed a new treatment strategy that targets autophagy to combat coronavirus disease-19 (COVID-19) (Yang and Shen, 2020). In addition, there has been some progress in targeting autophagy to protect against heavy metal poisoning. Previous studies demonstrated that activating autophagy and restoring autophagic flux are important mechanisms to alleviate the toxic injury caused by lead (Pb) or Cd (Song X. et al., 2017; Liu et al., 2019; Zhu et al., 2019; Gu et al., 2020). However, the detailed mechanisms involved in targeting autophagy require further exploration.

In the past few decades, researchers have explored the toxicity mechanism of heavy metals and its protective strategies. Based on the toxicity mechanism of heavy metals, many feasible protection strategies have been proposed, among which natural antioxidants have attracted much attention (Brzóska et al., 2016). Puerarin (Pue) is a natural antioxidant derived from plants (extracted from the roots of the traditional Chinese herbal medicine *Pueraria lobata*). Pue has a wide range of pharmacological effects, playing an important role in the treatment of cardiovascular diseases, neurodegenerative diseases, diabetes, and cancer (Wang et al., 2006; Zhang, 2019). In addition, Pue has positive effects in resisting cell injury induced by heavy metals. Pue can not only ameliorate Cd-induced adverse effects, but also can alleviate nickel- and Pb-induced cell injury (Liu et al., 2016; Song et al., 2016). Given its strong antioxidant capacity, the mechanisms by which Pue protect against heavy metal-induced cell injury mainly include anti-oxidation and anti-apoptosis (Liu et al., 2012; Song et al., 2016). Further research revealed the role of Pue in alleviating heavy metal poisoning by regulating autophagy. For example, Pue plays an important role in protecting against Pb-induced injury in primary rat proximal tubular (rPT) cells by activating autophagy and restoring autophagic flux (Song X. et al., 2017). A similar mechanism has also been reported in alleviating Cd-induced liver cell injury (Zhou et al., 2019). These studies have initially explored the role of targeting autophagy in Pue-mediated alleviation of heavy metal poisoning; however, the detailed molecular mechanisms are poorly understood. Therefore, the present study aimed to use the alpha mouse liver 12 (AML12) cell line as a model to further explore the detailed mechanism of Pue in alleviating Cd-induced hepatocyte injury by targeting autophagy, especially the two stages of fusion and degradation.

## Materials and Methods

### Chemicals and Reagents

Cadmium chloride was purchased from Sigma-Aldrich (202,908, St. Louis, MO, Unites States). Puerarin (purity >99%) was purchased from Selleck Chemicals (S2346, Houston, TX, Unites States). Dulbecco's modified Eagle’s Medium/Nutrient Mixture F-12 (DMEM/F-12) and insulin–transferrin–selenium-A supplement (ITS-A) were purchased from Gibco (Grand Island, NY, United States). The Dansylcadaverine (MDC) Kit was purchased from Solarbio (G0170, Beijing, China). Lyso-Tracker Red (LTR) was purchased from Beyotime (C1046, Shanghai, China). Bafilomycin A1 (Baf) and DQ-BSA-Red (Self-Quenched BODIPY FL Conjugate of bovine serum albumin) were purchased from Invitrogen (Carlsbad, CA, United States). All of the other materials were of analytical grade.

### Cell Culture and Treatment

The AML12 cell line was obtained from ATCC (Manassas, VA, United States) and cultured in DMEM/F-12 complete medium (containing 10% fetal bovine serum (FBS), 0.6% ITS-A, 100 U/ml penicillin, and 100 mg/ml streptomycin) at 37°C in the presence of 5% CO_2_ and saturated humidity. Replace complete medium with serum-free medium when treating cells. Cadmium chloride and Pue were dissolved in ultrapure water and dimethyl sulfoxide (DMSO) respectively. In this study, the final concentration of DMSO in the medium was less than 0.1%.

### Analysis of Cell Proliferation

The real-time cell analysis (RTCA) system (Roche Applied Science, Basel, Switzerland) and 5-Ethynyl-2′-deoxyuridine (EdU) Cell Proliferation Kit with Alexa Fluor 488 (C0071, Beyotime) were used in this study to determine the proliferation of AML12 cells. Briefly, for the RTCA system, AML12 cells were cultured in E-plates, and the cell index (CI) was monitored every 15 min. Then, different treatments were applied when the average CI reached approximately 2.5. The CI was finally presented as the Normalized cell index. For EdU staining, AML12 cells were seeded in 24-well plates and cultured to approximately 70% confluence, followed by treatment with various concentrations of Pue (50, 100, 200, and 400 μM) and/or 5 μM Cd for 12 h. After treatment with Pue and Cd, the cells were incubated in EdU working solution for 2 h, and then treated with 4% paraformaldehyde, 0.3% Triton X-100, Click Additive Solution, and Hoechst 33342, in that order, according to the manufacturer’s protocol. Finally, images were captured under a Leica DMIRB inverted microscope (DMI3000B, Leica, Wetzlar, Germany). Cells positive for EdU were counted as proliferative cells.

### Analysis of Cell Morphology

AML12 cells were seeded in 24-well plates and cultured to approximately 70% confluence, followed by treatment with 200 μM Pue and/or 5 μM Cd for 24 h. Then, images of cells were captured under the Leica DMIRB inverted microscope.

### Analysis Using Transmission Electron Microscopy

AML12 cells were seeded in 10-cm dishes and cultured to approximately 70% confluence, followed by treatment with 200 μM Pue and/or 5 μM Cd for 12 h. Then, the cells were fixed using 2.5% glutaraldehyde and osmium tetroxide, dehydrated in graded ethanol, soaked in Spurr resin, stained with uranium acetate and lead citrate, in that order. Finally, the cell ultrastructure and autophagosomes were observed under a transmission electron microscope (CM 100, Philips, Holland).

### MDC Staining

AML12 cells were seeded in 24-well plates and cultured to approximately 70% confluence, followed by treatment with 200 μM Pue and/or 5 μM Cd for 12 h. Treatment with Earle's balanced salt solution (EBSS) medium was used as the positive control group. Before staining with the MDC working solution, the cells were fixed with 4% paraformaldehyde. Finally, the fluorescence images were captured under a fluorescence microscope (TCS SP8 STED, Leica).

### Western Blotting Analysis

After treatment, the total proteins of cells were extracted for western blotting analysis. Briefly, after sonication and lysis in ice-cold Radioimmunoprecipitation assay (RIPA) lysis buffer, the cells were centrifuged at high speed at 4°C to harvest the supernatant. The protein concentration was determined using a Bicinchoninic acid protein assay kit (P0011, Beyotime), and samples were adjusted to the same protein level. Equivalent amounts of protein samples were separated by 5–12% SDS-PAGE gels, and transferred to polyvinylidene fluoride membranes. Then, the membranes were incubated with 5% nonfat milk for 2 hat room temperature, followed by incubation with primary antibodies diluted 1:1,000 in 5% bovine serum albumin (BSA) (dissolved in Tris-buffered saline with 0.05% Tween-20) overnight at 4°C. The primary antibodies used in this study were as follows: anti-microtubule associated protein 1 light chain 3 beta (LC3B) (L7543, Sigma, St. Louis, MO, United States), anti-p62/SQSTM1 (P0067, Sigma), anti-lysosomal associated membrane protein 2 (LAMP2) (L0068, Sigma), anti-cathepsin B (CTSB) (31718, Cell Signaling Technology, Boston, MA, United States), anti-Rab7 (9367, Cell Signaling Technology), and anti-β-actin (4970, Cell Signaling Technology). After incubation with the corresponding secondary antibodies, the membranes were imaged using a chemiluminescence imaging system (Tanon, Shanghai, China) with an enhanced chemiluminescence kit (New Cell and Molecular Biotech Co., Ltd., Suzhou, China). The gray values of the protein bands were analyzed using the ImageJ software (National Institutes of Health, Bethesda, MD, United States), and quantified relative to β-actin. All assays were performed at least three times.

### Analysis of Lysosomal Degradation Capacity

DQ-BSA-Red was used in this study to determine the effect of Pue and Cd on the lysosomal degradation capacity in AML12 cells. In brief, AML12 cells were seeded in confocal dishes and cultured to approximately 70% confluence. The cells were pretreated with 10 μg/ml DQ-BSA Red for 2 h, followed by treatment with 200 μM Pue and/or 5 μM Cd for 12 h. Finally, after staining with Hoechst 33342, the fluorescence images were captured rapidly under a fluorescence microscope (TCS SP8 STED, Leica).

### LTR Staining

After treatment with 200 μM Pue and/or 5 μM Cd for 12 h, the cells were incubated with LTR working solution at 37°C for 30 min, and then the fluorescence images were captured under the Leica DMIRB inverted microscope.

### Immunofluorescence Staining

AML12 cells (1.3 × 10^5^ cells per well) were seeded on the glass coverslips in 24-well plates for 12 h. After different treatments, double staining was performed as follows. First, after fixation with 4% paraformaldehyde, the cells were incubated separately with 0.5% Triton X-100 and 5% bovine serum albumin. Next, the cells were incubated with anti-LC3B antibodies diluted 1:200 in 5% BSA (L7543, Sigma), together with anti-LAMP2 antibodies diluted 1:100 in 5% BSA (sc-19991, Santa Cruz Biotechnology, Santa Cruz, CA, United States) overnight at 4°C. Then, the cells were incubated with secondary antibodies labeled with Alexa Fluor 488 and Cy3 (diluted 1:200 in 5% BSA) (Beyotime). Finally, after staining with 4′,6-diamidino-2-phenylindole (DAPI), the fluorescence images of the cells were observed under a fluorescence microscope (TCS SP8 STED, Leica).

### Infection and Analysis of StubRFP-SensGFP-LC3 Lentivirus

The stubRFP-sensGFP-LC3 (RFP, red fluorescent protein; GFP, green fluorescent protein) lentivirus was purchased from GeneChem Corporation (Shanghai, China). AML12 cells were seeded in 24-well plates and cultured to approximately 20% confluence, followed by co-incubation with stubRFP-sensGFP-LC3 lentivirus for 48 h according to the manufacturer’s protocols. Then, the positive cells were selected using 4 μg/ml puromycin (Solarbio) to establish stable cell lines. The cells marked with GFP-RFP-LC3 were seeded on glass coverslips in 24-well plates. At 70% confluence, the complete medium was replaced with serum-free medium, followed by treatment as required by the experiments. Then, the fluorescence images of LC3 puncta were viewed using a fluorescence microscope (TCS SP8 STED, Leica). The yellow LC3 puncta indicate autophagosomes and the red LC3 puncta indicate autophagolysosomes.

### Small Interfering RNA Transfection

The siRNA specific for *Rab7* was synthesized by RiboBio Corporation (Guangzhou, China). The siRNA sequence targeting the *Rab7* mRNA was follows: si-Rab7, 5′-CCA​TCA​AAC​TGG​ACA​AGA​A-3′. Briefly, to knockdown *Rab7*, AML12 cells were seeded in 6-well or 24-well plates. At 40% confluence, the cells were cultured in fresh medium without penicillin and streptomycin, together with transfection reagent (Polyplus-transfection, Illkirch, France) and 20 nM *Rab7* siRNA or negative control siRNA for 24 h, according to the manufacturer’s protocols. Then, the cells were collected and analyzed the knockdown efficiency using western blotting or were subjected to other treatments as required by the experiments.

### Statistical Analysis

Analyses of Data from at least three independent experiments were performed using one-way analysis of variance and Scheffe’s F test using GraphPad Prism 6 software (GraphPad Software Inc., La Jolla, CA, United States). The results are shown as the mean ± standard deviation (SD) and the *p <* 0.05 was considered statistically significant.

## Results

### Pue Alleviated Cd-Induced Injury in AML12 Cells

**FIGURE 1:**
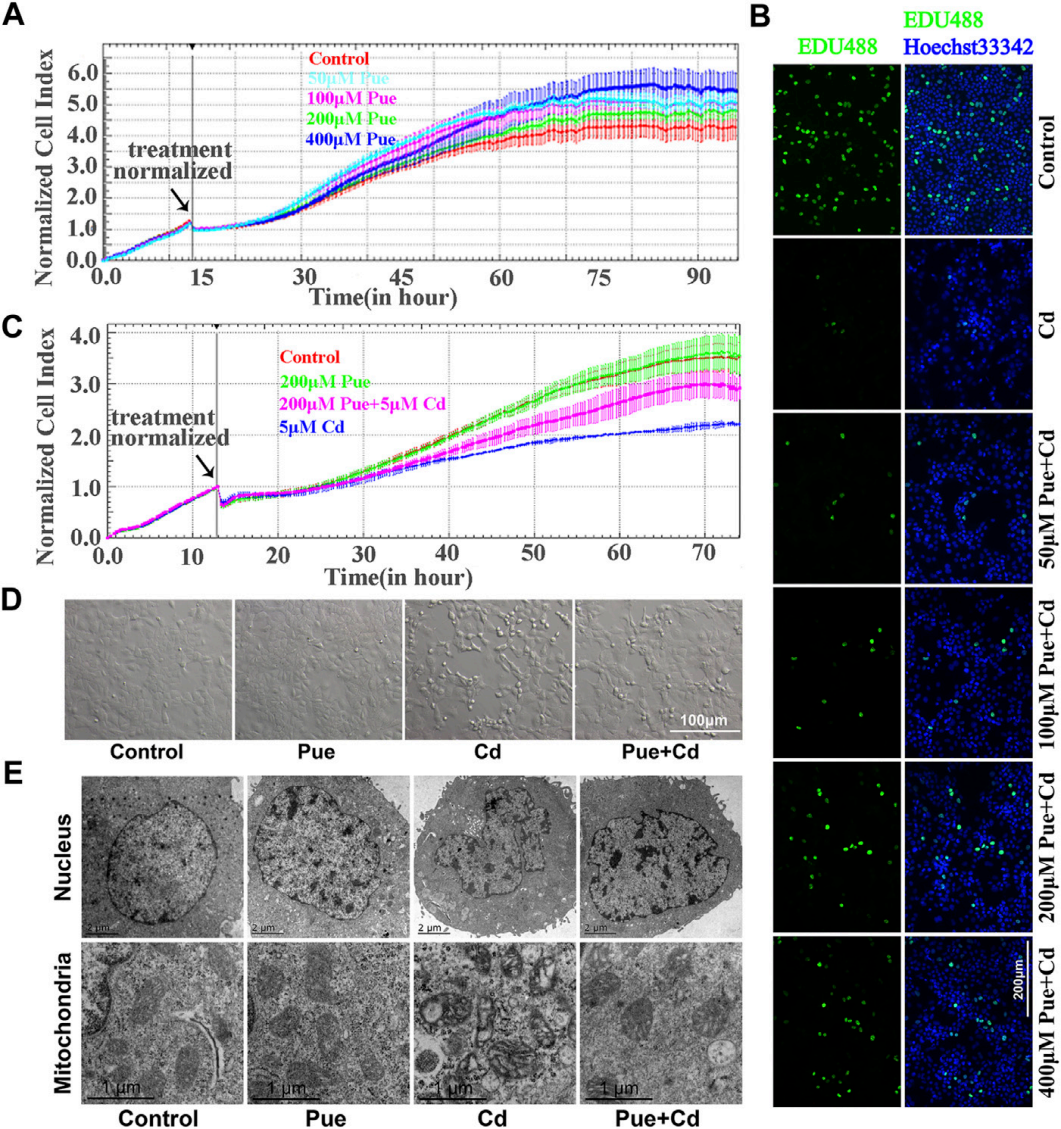
Pue alleviated Cd-induced injury in AML12 cells. **(A)** Effect of Pue at different concentrations (50, 100, 200, and 400 μM) on the CI in AML12 cells. Error bars represents the standard deviation (n = 3). **(B)** After treatment with Pue (50, 100, 200, and 400 μM) and/or 5 μM Cd for 24 h, cell proliferation was detected by EdU staining. Scale bar = 200 μm. **(C)** Effect of Pue and Cd, alone or in combination, on CI in AML12 cells. Error bars represents the standard deviation (n = 3). **(D)** Effect of Pue and Cd, alone or in combination, on cell morphology. Scale bar = 100 μm. **(E)** After treatment with Pue and Cd, alone or in combination, for 12 h, the changes in cell ultrastructure was analyzed under a transmission electron microscope and representative images are shown. Scale bar in nuclei = 2 μm; scale bar in mitochondria = 1 μm.

In the present study, the protective effect of Pue toward Cd-induced hepatocyte injury was confirmed mainly from the aspects of cell proliferation, cell morphology, and ultrastructure. First, referring to previous studies, different concentrations of Pue (50, 100, 200, and 400 μM) were tested, and the effect of Pue on cell proliferation in AML12 cells were determined by RTCA. The results showed that there was no significant difference in the CI of the 50–200 μM Pue treatment groups compared with the control group, except that the 400 μM Pue treatment group showed a slight increase (Figure 1A). Then, the effects of different concentrations of Pue (50, 100, 200, and 400 μM) in combination with 5 μM Cd on the proliferation of AML12 cells were detected by EdU staining. The results showed that the number of EdU-positive cells was significantly reduced in the Cd treatment group compared with that in the control group. However, the number of EdU-positive cells was increased after combined treatment with Pue compared with the Cd-alone treatment group, especially in the 200 or 400 μM Pue and Cd combined treatment group (Figure 1B). Considering the slight influence of 400 μM Pue on cell proliferation, the combination of 200 μM Pue with 5 μM Cd was selected to further verify the protective effect of Pue in Cd-exposed cells using the RTCA system. The results showed that the CI in the Cd treatment group decreased significantly compared with that in the control group. However, it increased in the Cd and Pue combined treatment group compared with that in the Cd-alone treatment group (Figure 1C). Finally, the same protective effect was also reflected in the changes in cell morphology and cell ultrastructure. The results are shown in Figures 1D,E. In the Cd-treated group, the cells became smaller and rounded, the nuclei shrank, and the mitochondria swelled and became vacuolated. However, in the combined treatment group, the above changes were alleviated. These results showed that Pue could effectively alleviate Cd-induced injury in AML12 cells.

### Pue Activated Autophagy and Alleviated Cd-Induced Accumulation of Autophagosomes in AML12 Cells

**FIGURE 2:**
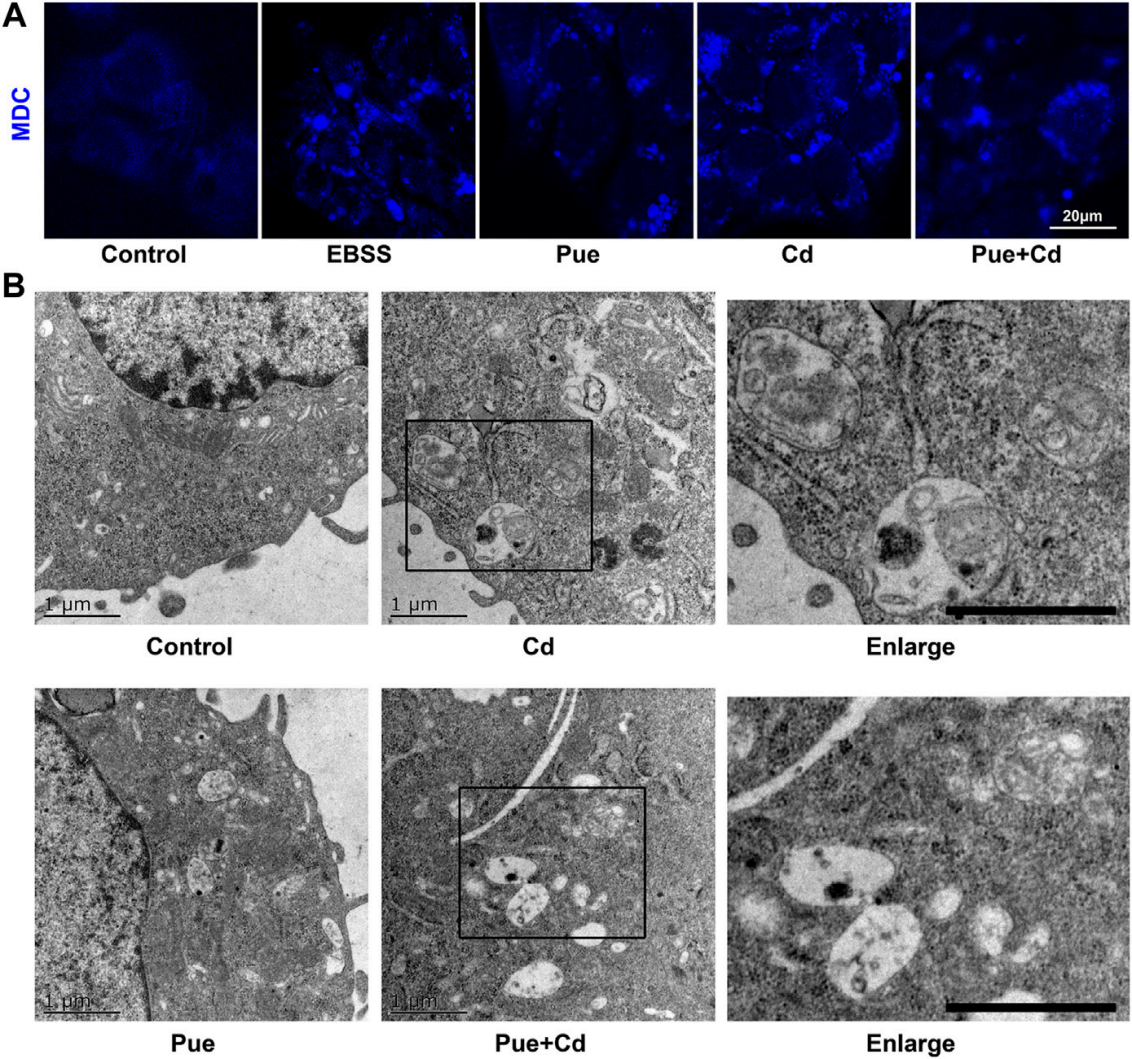
Pue activated autophagy and alleviated Cd-induced accumulation of autophagosomes in AML12 cells. **(A)** Cells were treated with Pue and/or Cd for 12 h, and then the autophagy levels were analyzed usisng MDC staining. Representative images of MDC staining are shown. Scale bar = 20 μm. **(B)** After treatment with Pue and Cd, alone or in combination, for 12 h, images of autophagosomes and autophagolysosomes were captured under a transmission electron microscope. Representative images of autophagosomes and autophagolysosomes are presented. Scale bar = 1 μm.

Next, we explored the possible mechanisms of the protective effect of Pue from the perspective of regulating autophagy. First, the changes in autophagy levels were preliminarily reflected by MDC staining (Figure 2A). Compared with that in the control group, the number of MDC fluorescent puncta increased significantly in the Cd and Pue alone or combined treatment group and the positive control group (EBSS-treatment group). However, the number of MDC fluorescent puncta in the Cd and Pue combined treatment group was reduced compared with that in the Cd-alone treatment group. Furthermore, transmission electron microscopy showed that a large number of autophagosomes with a double-layer membrane structure were accumulated in the cells treated with Cd alone. However, the number of autophagosomes decreased after combined treatment with Pue, while the number of autophagolysosomes, with a monolayer membrane structure, increased, and most of the materials contained in them were degraded (Figure 2B).

### Pue Alleviated Cd-Induced Autophagy Blockade in AML12 Cells

**FIGURE 3:**
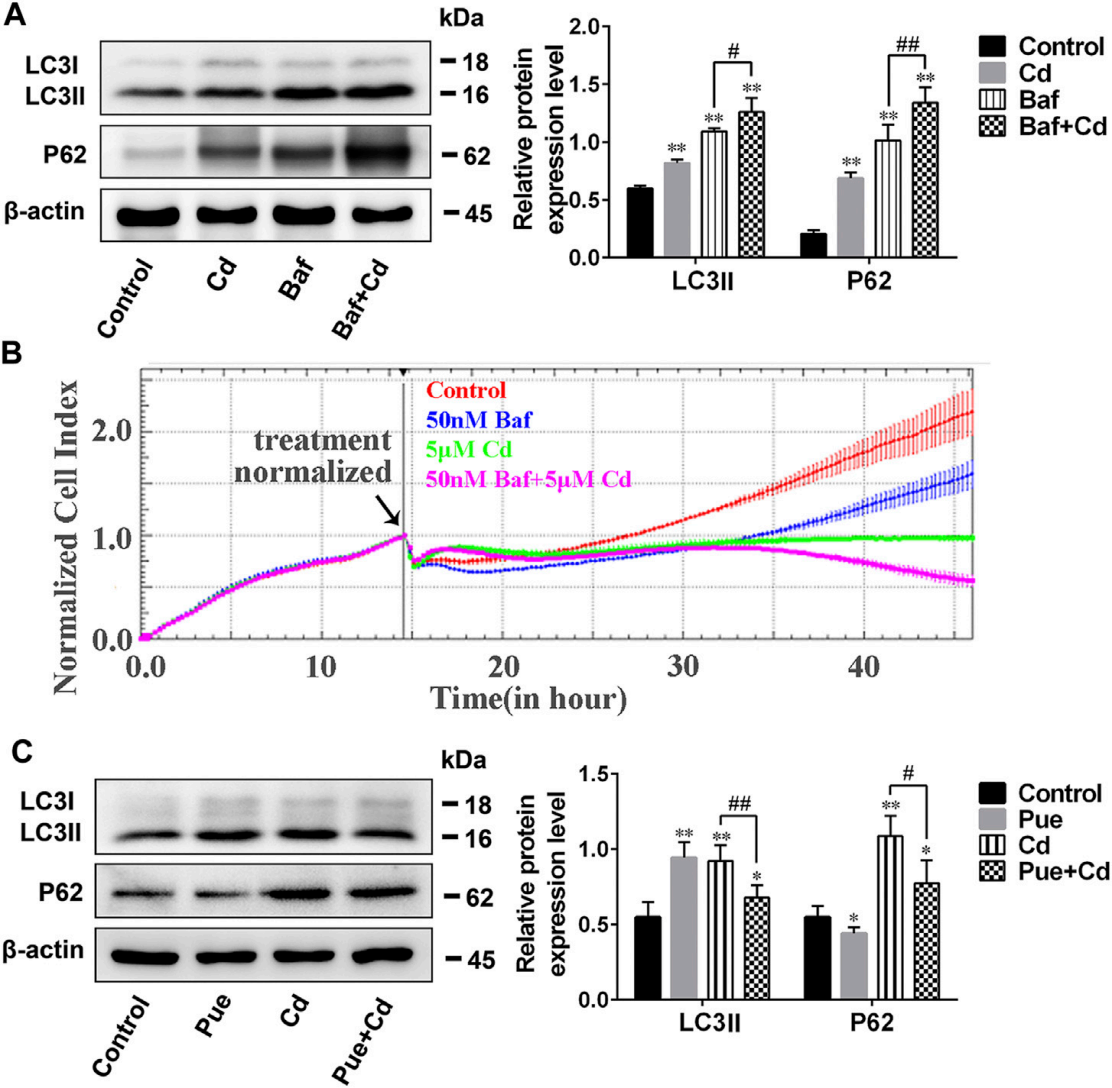
Pue alleviated Cd-induced autophagy blockade in AML12 cells. **(A)** Cells were treated with Cd and Baf, alone or in combination, for 12 h, and then the levels of LC3 and P62 were detected using western blotting to reflect the changes in autophagic flux. Data are shown as the mean ± SD (n = 4). Compared with the control group, ^**^ *p* < 0.01. Compared with the Baf-treatment group, ^#^ *p* < 0.05, ^##^ *p* < 0.01. **(B)** After treatment with Cd and/or Baf, the CI was monitored using RTCA to reflect the effect of autophagy blockade on Cd-induced cell injury. Error bars represents the standard deviation (n = 3). **(C)** After treatment with Pue and/or Cd for 12 h, cells were collected and the levels of LC3 and P62 were analyzed using western blotting. β-actin was used as the reference protein, and representative protein bands are shown. Data are shown as the mean ± standard deviation (SD) (n = 4). Compared with the control group, ^*^ *p* < 0.05, ^**^ *p* < 0.01. Compared with the Cd-treatment group, ^#^ *p* < 0.05, ^##^ *p* < 0.01.

To explore whether the protective effect of Pue is related to the alleviation of autophagy blockade, the effect of Pue and Cd on the autophagic flux in AML12 cells was monitored using western blotting. As shown in Figure 3A, after the combined treatment of Cd and Baf (a late-stage autophagy inhibitor), the levels of LC3II and P62 were further increased compared with those in the Baf-alone treatment group (*p* < 0.05 or *p* < 0.01), suggesting that autophagic flux was blocked. Next, the effect of autophagy blockade on cell proliferation was further verified using the RTCA system. The results showed that the CI in Cd-alone treatment group was significantly decreased compared with that in the control group. Notably, the CI in Cd and Baf combined treatment group decreased further compared with the that Cd-alone treatment group (Figure 3B). The above results confirmed that autophagy blockade is an important factor in Cd-induced cell injury. Then, the effect of Pue on the autophagic flux in Cd-exposed cells was detected using western blotting (Figure 3C). Compared with that in the control group, the relative level of LC3II in the Pue-alone treatment group increased significantly (*p* < 0.01), while the level of P62 decreased significantly (*p* < 0.05). Notably, the relative levels of LC3II and P62 in the Cd and Pue combined treatment group were higher than those in the control group, but significantly lower than those in the Cd-alone treatment group. This indicated that Pue could not only promote autophagy, but also alleviated Cd-induced autophagy blockage. Overall, the above results suggested that the protective effect of Pue in Cd-exposed cells is related to its alleviation of autophagy blockade.

### Pue and Cd Promoted Lysosomal Degradation by Triggering Lysosomal Activation in AML12 Cells

**FIGURE 4:**
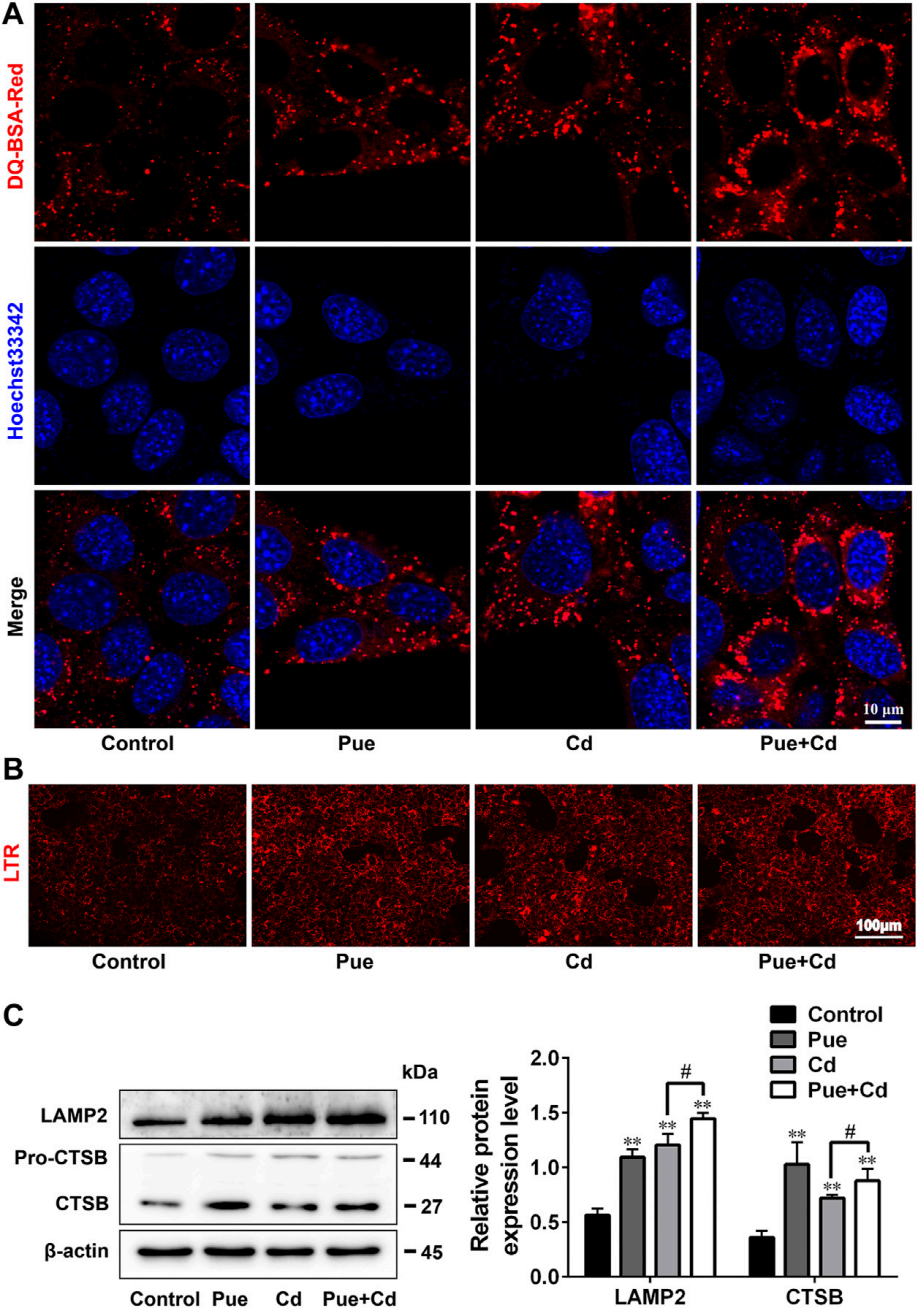
Pue and Cd promoted lysosomal degradation by triggering lysosomal activation in AML12 cells. **(A)** The effect of Pue and Cd on lysosomal degradation in AML12 cells was analyzed using DQ-BSA-Red staining. Representative fluorescence images are shown, and the red fluorescence indicates the fluorescent fragment released after DQ-BSA was degraded by the lysosome. Scale bar = 10 μm. **(B)** The effect of Cd and Pue in combination on the lysosomal acidity in AML12 cells was reflected by LTR staining. Scale bar = 100 μm. **(C)** Cells were treated with Pue and Cd, alone or in combination, for 12 h, and then the levels of LAMP2 and CTSB were examined using western blotting. Data are shown as the mean ± SD (n = 3). Compared with the control group, ^**^ *p* < 0.01. Compared with the Cd group, ^#^ *p* < 0.05.

Next, we explored the possible detailed mechanisms by which Pue alleviates autophagy blockade in Cd-exposed cells. First, the effect of Pue and Cd on lysosomal degradation was detected using the DQ-BSA assay. DQ-BSA will release fluorescence after being degraded by lysosomes, thus indirectly reflecting the degradation function of lysosomes (Li et al., 2015). As shown in Figure 4A, compared with the control group, the intensity and number of red fluorescent spots increased in the Pue- and Cd-alone treatment groups, and further increased in the co-treatment group. Then, the effects of Pue and Cd on lysosomal acidity and the expression of lysosomal-related proteins were detected by LTR staining and western blotting, respectively. After treatment with Pue and Cd alone or in combination, the fluorescence intensity of LTR and the levels of LAMP2 and CTSB increased significantly. Although there was no significant difference in the fluorescence intensity of LTR in the combined treatment group compared with the Cd alone treatment group, the levels of LAMP2 and CTSB further increased (Figures 4B,C). The above results suggest that both Pue and Cd could promote lysosomal degradation, which might be related to triggering lysosomal activation.

### Pue Restored Autophagosome-Lysosome Fusion and the Expression Levels of Rab7 in Cd-Exposed AML12 Cells

**FIGURE 5:**
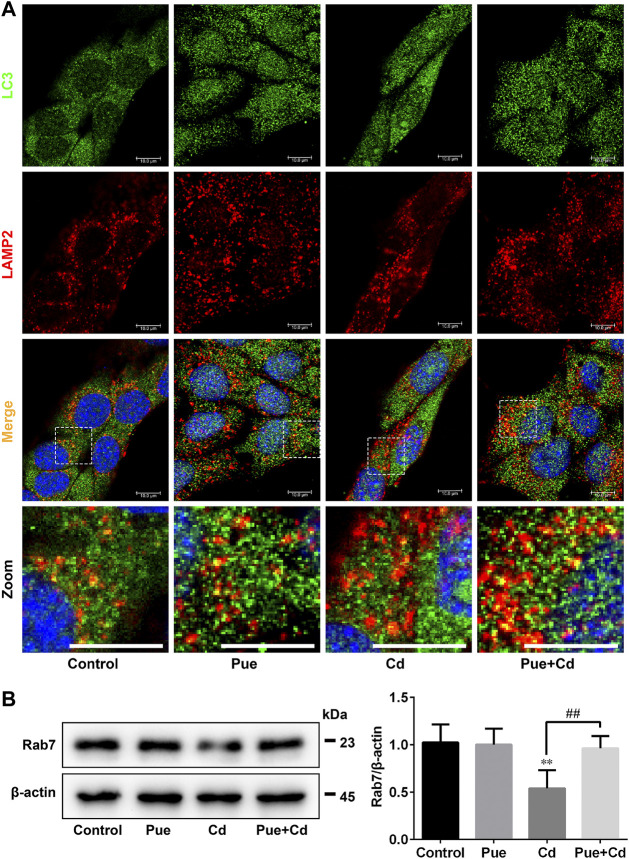
Pue restored autophagosome–lysosome fusion and the expression levels of Rab7 in Cd-exposed AML12 cells. Cells were treated with Pue and Cd for 12 h. **(A)** The co-localization of LC3 with LAMP2 was examined by immunofluorescence staining to reflect the fusion of autophagosomes and lysosomes. Representative fluorescence images and their partial enlarged images are shown; the yellow fluorescence puncta indicate the fused autophagosomes and lysosomes. Scale bar = 10 μm. **(B)** The levels of Rab7 were analyzed using western blotting. Data are shown as the mean ± SD (n = 4). Compared with the control group, ^**^ *p* < 0.01. Compared with the Cd group, ^##^ *p* < 0.01.

We further explored the detailed mechanisms by which Pue alleviated autophagy blockade in Cd-exposed cells by assessing the regulation of autophagosome-lysosome fusion. Double immunofluorescence staining of LC3 and LAMP2 was used to analyze autophagosome-lysosome fusion, in which yellow puncta indicated the occurrence of autophagosome-lysosome fusion. The results showed that the co-localization of LC3 and LAMP2 was significantly reduced after Cd-alone treatment, but was significantly increased after the combined treatment compared with that in the Cd-alone treatment (Figure 5A). Next, we detected the level of Rab7, a key protein involved in autophagosome-lysosome fusion, by western blotting. The results showed that Rab7 levels were downregulated significantly after exposure to Cd (*p* < 0.01), and restored after co-treatment with Pue (*p* < 0.01) (Figure 5B). These results indicated that Pue alleviated Cd-induced autophagy blockade by restoring autophagosome-lysosome fusion, and the mechanism was related to the restoration of the protein levels of Rab7.

### Rab7 Played an Important Role in Pue-Alleviated Cd-Induced Autophagy Blockade

**FIGURE 6:**
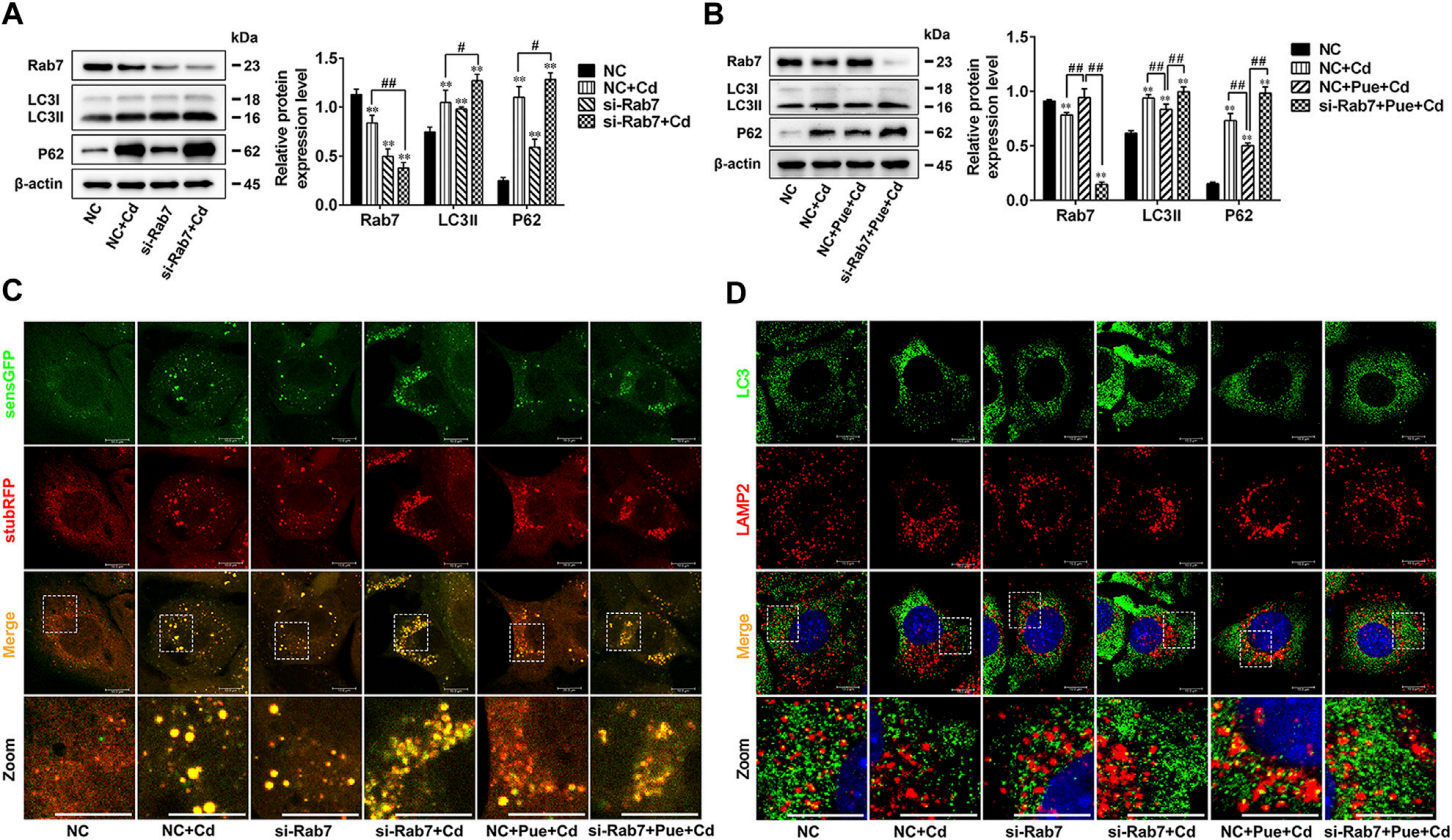
Rab7 played an important role in Pue-alleviated Cd-induced autophagy blockade in AML12 cells. Based on treatment with a Rab7 siRNA or NC siRNA for 24 h. **(A)** Cells were treated with or without Cd for 12 h, **(B)** cells were treated with Pue and/or Cd for 12 h, and then the levels of Rab7, LC3, and P62 were analyzed using western blotting. Data are shown as the mean ± SD (n = 3). Compared with the NC group, ^**^ *p* < 0.01. Compared with the NC + Cd group or NC + Pue + Cd group, ^#^ *p* < 0.05, ^##^ *p* < 0.01. **(C)** Cells stably expressing stubRFP-sensGFP-LC3 were treated with Rab7 siRNA or NC siRNA for 24 h, followed by treatment with Pue and/or Cd for 12 h, and then the LC3 puncta were captured under a fluorescence microscope. Representative images of LC3 puncta and their partial enlarged pictures are shown. Scale bar = 10 μm. **(D)** After treated with Rab7 siRNA or NC siRNA for 24 h, cells were treated with Pue and/or Cd for 12 h, and then the co-localization of LC3 with LAMP2 was analyzed using immunofluorescence staining. Representative pictures and their partial enlarged pictures are displayed. Scale bar = 10 μm.

To further verify the key role of Rab7 in Pue-mitigated Cd-induced inhibition of autophagosome-lysosome fusion, the *Rab7* siRNA was employed in the cell model. Western blot analysis showed that, compared with that in the negative control (NC) group, knocking down *Rab7* resulted in a significant increase in the levels of LC3II and P62, which was consistent with the results in NC + Cd group (*p* < 0.01) (Figure 6A). The results of RFP-GFP-LC3 analysis showed that, compared with that in the NC group, knocking down Rab7 resulted in an increase in the accumulation of yellow LC3 puncta (autophagosomes), which was consistent with the results of the NC + Cd group. Compared with the NC + Cd group, the accumulation of yellow LC3 puncta further increased after Cd and si-Rab7 combined treatment (Figure 6C). These results were consistent with the results of the protein level changes in Figure 6A, indicating that the Cd-induced autophagy blockade was indeed related to the downregulation of Rab7. In addition, immunofluorescence staining showed that the co-localization of LC3 and LAMP2 was reduced in both the si-Rab7 group and the si-Rab7+Cd group, indicating that Cd inhibited the autophagosome-lysosome fusion by downregulating Rab7 levels (Figure 6D). Moreover, in the NC + Pue + Cd group, the protein level changes were consistent with the results of the RFP-GFP-LC3 analysis compared with those in the NC + Cd group, which showed that the autophagy blockade was alleviated (Figures 6B,C). Meanwhile, immunofluorescence staining also showed that autophagosome-lysosome fusion was restored (Figure 6D). However, compared with that in the NC + Pue + Cd group, after knocking down *Rab7*, the level of LC3II and P62 increased significantly (*p* < 0.01) (Figure 6B), the yellow LC3 puncta increased and the red LC3 puncta (autophagolysosomes) decreased (Figure 6C), similarly, the co-localization of LC3 and LAMP2 decreased (Figure 6D). These results indicated that after knocking down *Rab7*, the protective effects of Pue in restoring autophagosome-lysosome fusion and alleviating autophagy blockade were inhibited.

## Discussion

The preparation and genetic manipulation of primary hepatocytes is difficult; therefore, in the present study, we chose AML12 cells to construct a Cd-induced hepatocyte injury model. AML12 cells are immortalized hepatocytes that retain typical hepatocyte characteristics, and their response to autophagy might be close to that of normal hepatocytes (Wu et al., 1994; Wang et al., 2017). Therefore, AML12 cells were a suitable cell model for this study. Pue is a natural antioxidant derived from plants. Based on its powerful anti-oxidation, anti-apoptosis, and autophagy regulating effects, the positive effects of Pue in protecting against heavy metal induced cell injury have been reported in many articles. Previous studies showed that 50–200 μM Pue could alleviate Pb- or Cd-induced cell injury in rPT cells, which provided a reference for choosing the working concentration range of Pue in this study (Liu et al., 2014; Song et al., 2016). After screening and considering the results of our previous study (Zou et al., 2020), 200 μM Pue and 5 μM Cd were finally selected for the subsequent mechanistic research described in this study. In addition, in a recently published article, the protective effect of Pue in Cd-exposed hepatocytes was reported in terms of cell viability and apoptosis (Zhou et al., 2019). In the present study, the protective effect of Pue was further confirmed according to changes in cell proliferation, cell morphology, and cell ultrastructures using RTCA, EdU staining, and transmission electron microscopy.

Autophagy is a process by which “trash” (such as damaged organelles and misfolded proteins) in cells is digested and recycled by lysosomes (Cuervo and Macian, 2012; Schneider and Cuervo, 2014). Under physiological conditions, a low level of autophagy is maintained in cells, which is considered to be a quality control mechanism that is especially important for cell homeostasis (Ueno and Komatsu, 2017). As a survival mechanism, autophagy is considered as a potential therapeutic target for many diseases, such as metabolic syndrome diseases, neurodegenerative disorders, autoimmune diseases, and cancer (Condello et al., 2019). In addition, the stage-specific regulation of autophagy is a new intervention method for liver disease (Wang, 2015). In the study of the toxicity mechanism of heavy metals, it was found that during heavy metal exposure, autophagy was activated to fight the induced stress (Wang et al., 2013). However, under continuous heavy metal exposure, autophagic flux was disrupted (the fusion or degradation stage was blocked), resulting in the accumulation of autophagosomes that could not be degraded and recycled (Liu F. et al., 2017; Song X.-B. et al., 2017; Zou et al., 2020). These reports prompted us to study the role of targeting autophagy in protecting against heavy metal-induced cell injury.

Previous studies indicated that activating autophagy can alleviate Cd-induced cell damage (Zou et al., 2015; Liu G. et al., 2017). Pue can activate autophagy via a mechanism related to the inhibition of mammalian target of rapamycin (mTOR). A study documented that in a rat cardiac hypertrophy model, Pue activated autophagy to alleviate cardiomyocyte hypertrophy and apoptosis by activating the phosphorylation of 5′AMP-activated protein kinase (AMPK) and inhibiting mTOR signaling, and this protective effect was blocked by the autophagy inhibitor, 3-methyladenine (Liu et al., 2015). This indicated that the protective effect of Pue is related to the activation of autophagy. Similarly, Pue alleviated liver damage in an ethanol-induced liver injury model by activating autophagy via AMPK/mTOR-mediated signaling (Noh et al., 2011). In the present study, Pue’s effect of activating autophagy was confirmed by MDC staining, observation of autophagosomes, and analysis of autophagy marker proteins. Our results were consistent with those of a recently published article (Zhou et al., 2019). However, the response of autophagy activation to Pue has been reported differently in other studies. Studies have found that in rPT cells, 100 μM Pue could alleviate Pb- or Cd-induced autophagic blockade, but had no obvious effect on activating autophagy (Song X. et al., 2017; Wang et al., 2019). These differences might be related to the different cell models and the dosages of Pue used. In addition, our results showed that, compared with the Cd group, the combined treatment of Pue and Cd reduced the overall autophagy level and alleviated the accumulation of autophagosomes. This suggested that Pue not only activates autophagy, but also participates in the regulation of autophagic flux.

Autophagic flux refers to the rate of cargo degradation via autophagy (Lumkwana et al., 2017). LC3 and P62 are the marker proteins of autophagic flux. LC3 reflects the overall level of autophagy, while P62 reflects the overall degradation produced by autophagy. Inhibition of autophagy at the later phases (the autophagosome-lysosome fusion phase or the autophagolysosome degradation phase) would lead to increased levels of LC3 and P62 (Yin et al., 2017; Yoshii and Mizushima, 2017). In addition, the analysis of GFP-RFP-LC3 fluorescence puncta is another commonly used method to monitor autophagic flux (Yoshii and Mizushima, 2017). Blockade of autophagic flux leads to autophagy dysfunction, which is fatal for cell survival (Singh et al., 2014). Autophagy blockade is associated with many diseases, including neurodegenerative diseases, cardiovascular diseases, cancer, and liver diseases. For these diseases, the regulation of autophagic flux is an important target for treatment (Morel et al., 2017; Allaire et al., 2019). Previous studies have indicated that autophagy blockade is an important mechanism in the toxicity of heavy metals (Song X.-B. et al., 2017; Zou et al., 2020). Alleviating autophagy blockade can ameliorate heavy metal-induced cell injury. Recent studies have found that the restoration of autophagic flux plays an important role in Pue-mediated alleviation of Pb- or Cd-induced rPT cell injury (Song X. et al., 2017; Wang et al., 2019). In this study, Cd-induced destruction of autophagic flux was further verified by analyzing the levels of LC3 and P62. Subsequent RTCA analysis showed that blockade of autophagosome clearance exacerbated the damage to Cd-exposed cells. However, Pue alleviated Cd-induced autophagy blockade, which was consistent with the results of a recently published article (Zhou et al., 2019). Therefore, our findings indicated that Pue-mediated alleviation of Cd-induced hepatocyte injury was related to the mitigation of autophagy blockade.

Blockade of autophagosome-lysosome fusion and lysosomal dysfunction are the main reasons for autophagy blockade. Studies have documented that in rPT cells, Cd induces autophagy blockade by inhibiting both autophagosome-lysosome fusion and autophagosome degradation, and Pue can alleviate autophagy blockade by restoring autophagic degradation (Liu F. et al., 2017; Wang et al., 2019). However, autophagy is an orderly and dynamic process, and from the formation of autophagosomes to the final degradation, each phase is strictly regulated by proteins encoded by autophagy-related genes (Al-Bari and Xu, 2020). The degradation of autophagosomes requires fusion with lysosomes; therefore, we inferred that to alleviate autophagy blockade, Pue might target factors in both the degradation phase and the fusion phase. This inference was confirmed by the results of the present study. Our results indicated that Pue alleviated Cd-induced autophagy blockade by restoring autophagosome-lysosome fusion in AML12 cells. Interestingly, in this process, Cd and Pue both triggered lysosomal activation and promoted lysosomal degradation. The explanation for this phenomenon, might be that in addition to the activation of autophagy, lysosomal activation is also be related to the changes in lysosomal adaptation triggered by lysosomal stress (Lu et al., 2017).

The fusion of autophagosomes and lysosomes occurs in an orderly manner via complex molecular regulation. Briefly, after autophagosomes are formed, they are transported along microtubules to lysosomes, mediated by dynein. Then, autophagosomes and lysosomes are tethered together via tethering factors. Finally, fusion is triggered under the mediation of soluble N-ethylmaleimide-sensitive factor attachment protein receptor (SNARE) complexes (Yu et al., 2018). Tethering factors, such as homotypic fusion and protein sorting (HOPS) complex, Rab7, and some adaptors, act as bridges in autophagosome-lysosome fusion (Ganley, 2013; Yu et al., 2018). Among them, Rab7 is a key regulatory factor and is required for autophagosome-lysosome fusion. In addition, Rab7 is a multifunctional autophagy regulatory factor, involved in the maturation, transportation, and fusion of autophagosomes, and thus is a potential therapeutic target for diseases (Gutierrez et al., 2004; Hyttinen et al., 2013; Wen et al., 2017). There is evidence that targeting Rab7 to regulate autophagy is a feasible strategy in disease treatment. A study indicated that in an acute liver injury model, upregulating Rab7 to maintain autophagy is essential for simvastatin-mediated alleviation of liver injury (Guixé-Muntet et al., 2017). In addition, previous studies have reported that the inhibition of autophagosome-lysosome fusion by Cd is related to the downregulation of Rab7 and its recruitment to autophagosomes (Liu F. et al., 2017; Zou et al., 2020). Therefore, we hypothesized that Rab7 is an important target for Pue-mediated restoration of autophagosome-lysosome fusion. This hypothesis was supported by the results of the current study, which showed that Pue restored Rab7 protein expression levels in Cd-exposed hepatocytes (Figure 5B). In addition, we evaluated the correlation between autophagosome-lysosome fusion and Rab7 by knocking down *Rab7* expression, which further confirmed that Rab7 is an important target for Pue-mediated regulation of autophagy via restoring autophagosome-lysosome fusion.

In summary, we revealed the detailed mechanism by which Pue targets autophagy to relieve Cd-induced hepatocyte injury, providing a new avenue for the study of targeting autophagy strategies to protect against heavy metal poisoning. Our results proved that Pue has multiple targets in the process of alleviating Cd-induced hepatocyte injury by targeting autophagy, involving the activation of autophagy and the alleviation of autophagy blockade. In addition, Pue restored the fusion of autophagosomes and lysosomes by restoring Rab7 protein expression levels, thereby alleviating Cd-induced autophagy blockade in hepatocytes. Consequently, we believe that Rab7 is an important target through which Pue regulates autophagy to alleviate the hepatotoxicity of Cd.

## Data Availability

The original contributions presented in the study are included in the article/Supplementary files, further inquiries can be directed to the corresponding author.

## Abbreviations

AML12, alpha mouse liver 12; Baf, bafilomycin A1; BSA, bovine serum albumin; Cd, cadmium; CI, cell index; COVID-19, coronavirus disease-19; CTSB, cathepsin B; DAPI, 4′,6-diamidino-2-phenylindole; DQ-BSA-Red, self-quenched BODIPY FL conjugate of BSA (Red); EdU, Ethynyl-2′-deoxyuridine; HOPS, homotypic fusion and protein sorting; ITS-A, insulin–transferrin–selenium-A supplement; LAMP2, lysosomal-associated membrane protein 2; LTR, Lyso-Tracker Red; MDC, dansylcadaverine; NC, negative control; Pb, lead; Pue, puerarin; RIPA, radioimmunoprecipitation assay; RTCA, real-time cell analysis; rPT, rat proximal tubular; SD, standard deviation; SNARE, soluble N-ethylmaleimide-sensitive factor attachment protein receptor.
