# Supplementary material for: Puerarin Restores Autophagosome-Lysosome Fusion to Alleviate Cadmium-Induced Autophagy Blockade via Restoring the Expression of Rab7 in Hepatocytes
Source: Front Pharmacol. 2021 Apr 14;12:632825. doi: 10.3389/fphar.2021.632825 (PMC8079953; doi:10.3389/fphar.2021.632825)
Supplement: Supplementary file 1 [file datasheet1.pdf]

Supplementary Material

1 Supplementary Data

Supplementary Material should be uploaded separately on submission. Please include any supplementary data, figures and/or tables. All supplementary files are deposited to FigShare for permanent storage and receive a DOI.

Supplementary material is not typeset so please ensure that all information is clearly presented, the appropriate caption is included in the file and not in the manuscript, and that the style conforms to the rest of the article. To avoid discrepancies between the published article and the supplementary material, please do not add the title, author list, affiliations or correspondence in the supplementary files.

2 Supplementary Figures and Tables

For more information on Supplementary Material and for details on the different file types accepted, please see here. Figures, tables, and images will be published under a Creative Commons CC-BY licence and permission must be obtained for use of copyrighted material from other sources (including re-published/adapted/modified/partial figures and images from the internet). It is the responsibility of the authors to acquire the licenses, to follow any citation instructions requested by third-party rights holders, and cover any supplementary charges.

2.1 Supplementary Figures

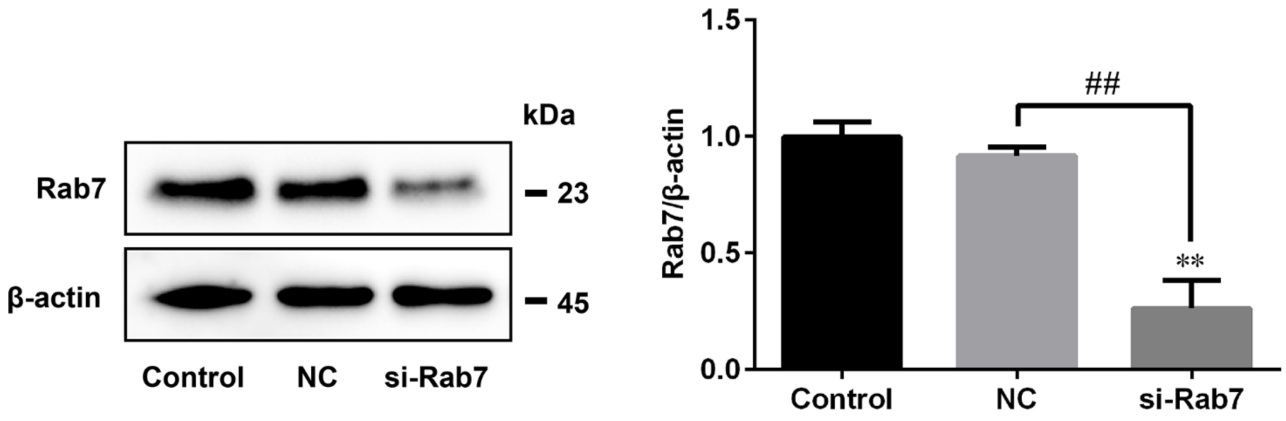

Supplementary Figure 1. The expression levels of Rab7 were silenced by siRab7 in AML12 cells. AML12 cells were treated with 20 nM Rab7 siRNA or negative control siRNA for 24 h. Then, the cells were collected and analyzed the knockdown efficiency using western blotting. Data are shown as the mean ± SD (n =3). Compared with the control group, $^{**}P < 0.01$. Compared with the NC group, $^{\#\#}P <$ 0.01.
